# Long-Term Results of Fixed High-Dose I-131 Treatment for Toxic Nodular Goiter: Higher Euthyroidism Rates in Geriatric Patients

**DOI:** 10.4274/mirt.57060

**Published:** 2015-11-02

**Authors:** Gül Ege Aktaş, Halil Turgut Turoğlu, Tanju Yusuf Erdil, Sabahat İnanır, Fuat Dede

**Affiliations:** 1 Trakya University Faculty of Medicine, Department of Nuclear Medicine, Edirne, Turkey; 2 Marmara University Faculty of Medicine, Department of Nuclear Medicine, İstanbul, Turkey

**Keywords:** Nodular goiter, I-131, geriatrics, Hyperthyroidism

## Abstract

**Objective:**

Geriatric patient population has special importance due to particular challenges. In addition to the increase in incidence of toxic nodular goiter (TNG) with age, it has a high incidence in the regions of low-medium iodine intake such as in our country. The aim of this study was to evaluate the overall outcome of high fixed dose radioiodine (RAI) therapy, and investigate the particular differences in the geriatric patient population.

**Methods:**

One hundred and three TNG patients treated with high dose I-131 (370-740 MBq) were retrospectively reviewed. The baseline characteristics; age, gender, scintigraphic patterns and thyroid function tests before and after treatment, as well as follow-up, duration of antithyroid drug (ATD) medication and achievement of euthyroid or hypothyroid state were evaluated. The patient population was divided into two groups as those=>65 years and those who were younger, in order to assess the effect of age.

**Results:**

Treatment success was 90% with single dose RAI therapy. Hyperthyroidism was treated in 7±7, 2 months after RAI administration. At the end of the first year, overall hypothyroidism rate was 30% and euthyroid state was achieved in 70% of patients. Age was found to be the only statistically significant variable effecting outcome. A higher ratio of euthyroidism was achieved in the geriatric patient population.

**Conclusion:**

High fixed dose I-131 treatment should be preferred in geriatric TNG patients in order to treat persistent hyperthyroidism rapidly. The result of this study suggests that high fixed dose RAI therapy is a successful modality in treating TNG, and high rates of euthyroidism can be achieved in geriatric patients.

## Abstract (tr)

**Amaç:**

Geriatrik hasta grubu kendine has özellikleri, acil ve zorlu durumları olan bir popülasyondur. Toksik nodüler guatr (TNG) sıklığı yaşla beraber artmaktadır, bununla birlikte ülkemiz gibi düşük iyot alımı bölgelerindeki sıklığı normal sıklığının daha da üzerindedir. Altmış beş yaş üzerinde ülkemizde bu oran %37,5 olarak bildirilmektedir. Bu geriye dönük çalışmada, yüksek sabit doz radyoaktif iyot (RAİ) uygulamasının sonuçlarını bütünüyle değerlendirmek ve özellikle geriatrik hasta grubunun sonuçlarında bir farklılık olup olmadığını araştırmak amaçlanmıştır.

**Yöntem:**

TNG nedeniyle, yüksek doz I-131 (370-740 MBq) tedavisi almış 103 hastanın takip dosyaları incelendi. Yaş, cinsiyet, sintigrafik bulgular, tedavi öncesi ve sonrası takip tiroid fonksiyon testleri, antitiroid ilaç tedavisinin süresi, ötiroidi veya hipotiroidi durumunun gelişimi değerlendirildi. Geriatrik hasta grubunun olası farklı sonuçlarını daha detaylı değerlendirmek amacıyla; 65 yaş ve üzeri hasta popülasyonu ayrıca ele alınarak, 65 yaş altı grubunun sonuçları ile karşılaştırıldı.

**Bulgular:**

Tek doz RAİ uygulaması sonucunda tedavi etkinliği %90 olarak bulundu. Hipertiroidi ortalama 7±7, 2 ayda tedavi edildi. Bir yılın sonunda, ötiroidi oranı %70, hipotiroidi oranı ise %30 olarak hesaplandı. İncelenen faktörler arasında yalnızca yaşın tedavi sonuçlarına anlamlı etkisi belirlendi. Geriatrik hasta grubunda daha yüksek oranda ötiroidi sağlandı.

**Sonuç:**

Sistemik hastalıkların sıklığı nedeniyle hızlı tedavi gerektiren geriatrik hasta grubunda, sabit yüksek doz I-131 uygulaması öncelikle tercih edilmesi gereken yöntemdir. Sabit yüksek doz RAİ ile TNG tedavisi, başarı oranı yüksek, hızlı ve etkin bir tedavidir ve geriatrik hasta gurubunda daha yüksek ötiroidi oranları elde edilebilir.

## INTRODUCTION

Plummer’s disease usually affects individuals over 50 years of age (1). On the other hand, 10-15% of hyperthyroid patients are over 60 years old (2). While the incidence of toxic nodular goiter (TNG) increases with age, it has a high incidence in the regions of low-medium iodine intake, like our country (3,4,5).

In the literature, usually the calculated and low fixed dose regimens of radioiodine (RAI) therapy are being discussed and compared in achieving high rates of euthyroidism while decreasing the total absorbed dose (6,7).

High rates of euthyrodism are expected after treatment of TNG with radiodine due to the relatively radioresistant nature of the nodules and the probable existence of different amounts of underlying suppressed thyroid tissue (8,9). Based on this fact and the need for rapid treatment of hyperthyroidism in geriatric patients, high fixed dose I-131 treatment regimen gained attraction. In addition, iodine uptake of the thyroid gland decreases with aging, therefore, a high rate of euthyroidism can be achieved in geriatric patients with treatment. In light of these data, the effectiveness and outcome of high dose RAI treatment of TNG patients and the possible difference in outcome in the geriatric group were evaluated in this study.

## MATERIALS AND METHODS

### Patients

The records of 177 TNG patients who were treated with I-131 in our department between 1996-2006 were retrospectively reviewed. Patients who had thyroid surgery before RAI therapy, who received RAI in another center before our treatment, and those who were lost to follow-up were excluded. The study group consisted of 103 TNG (14 toxic adenoma and 89 toxic multinodular goiter) patients, aged between 21-94 years old (mean: 63±12 years), including 44 women and 59 men, who were followed up properly for at least 1 year (Table 1).

Within the study population, 17 patients were not taking antithyroid drug (ATD) treatment because of liver dysfunction, five of which had liver insufficiency. One patient had schizoaffective disorder and did not comply with the medication and another had allergic reaction with ATD.

### Methods

The baseline characteristics including age, gender, scintigraphic evaluation, thyroid function tests before and after treatment including follow up, duration of ATD medication before therapy, achievement of euthyroid or hypothyroid state were reviewed. The factors that could affect the outcome of RAI therapy were assessed. The patient population was divided into two groups as those =>65 years and those who were younger in order to investigate the effect of age and to evaluate a possible difference in geriatric patient population’s outcome.

### Radioiodine Treatment Protocol

The patients who were referred to RAI therapy consisted of cases with nodule or nodules smaller than 4 cm in diameter and those with surgical contraindications. Fine needle aspiration biopsy was performed to all patients with suspicious nodules. Tc-99m thyroid scintigraphy was performed to all patients before treatment. A group meeting of Nuclear Medicine specialists on thyroid diseases was held properly in our department. All the patients were evaluated according to their scintigraphic and ultrasonographic findings, as well as results of thyroid palpation, thyroid function tests and thyroid autoantibodies. Patient age, co-morbidities especially like cardiovascular urgencies and atrial fibrillation were taken into consideration. A RAI dose between 370-740 MBq was determined empirically according to these findings. All the patients were informed about the disease and RAI treatment protocols including possible outcomes.

Intake of iodine rich food and contrast enhanced imaging protocols were avoided. ATD medication was discontinued 3 days before treatment, and the patients fasted for 6 hours before RAI therapy.

RAI was administered orally in the capsule form. The same ATD medication dose that patients were using -if the patient was on treatment- was restarted 3 days after RAI administration. The dose was reduced by half at the fourth post-RAI treatment week. The patients were followed-up by regular thyroid function test evaluations beginning from the second month after treatment (at 3-6 months intervals).

Thyroid function tests before and after therapy, and during follow-up were performed by chemiluminescence method, using E-170 modular (Roche-Hitachi) analyzer.

### Outcome

Treatment success was defined as achievement of euthyroid or hypothyroid state without ATD medication. Persistent or recurrent hyperthyroidism after 1 year of follow up was accepted as treatment failure.

### Statistical Analysis

The numerical data of the groups were tested with t-test and Mann-Whitney U test. Categorical data were evaluated with chi-square test. A p value ≤0.05 was accepted as statistically significant.

## RESULTS

One hundred and three TNG (14 toxic adenoma and 89 toxic multinodular goiter) patients (44 men and 59 women) received high fixed dose I-131 treatment of 370-740 MBq (10-20 mCi, mean: 13.78±2.91 mCi). The mean follow-up duration of patients was 44.3±26.2 months. Hyperthyroidism was treated in 7±7.2 months after RAI administration. The mean duration for appearance of hypothyroidism after therapy was found as 11.6±11.9 months. General characteristics of the patient population were summarized in Table 2.

Our treatment success was 90% with a single dose RAI therapy; only 10 patients required a second RAI dose, and hyperthyroidism was treated in all patients. At the end of the first year after RAI therapy, the overall hypothyroidism rate was 30% and a euthyroid state was achieved in 70% of patients. Complete follow-up records for four years were available in 63 patients. Only 40 patients were followed-up for more than four years. The rate of hypothyroidism increased to 37%, 40% and 42% on the second, third and fourth years post therapy, respectively.

Variables were analyzed in terms of their effect on the overall outcome, achievement of euthyroid state or development of hypothyroidism. Patient’s gender and scintigraphic pattern had no statistical effect on the outcome (Table 3). Only the effect of age was found to be statistically significant on outcome in univariate analysis, among multiple factors. Mean age of euthyroid patients was found to be significantly higher than those who were not (Table 4).

We compared patients’ characteristics by classifying them into two groups according to their age: group 1, representing patients under 65 years of age (n=51) and group 2, representing the ones equal to or older than 65 years old (n=52). There was no statistical difference between the distribution of scintigraphic patterns and gender between group 1 and group 2 (p=0.72 and p=0.22 respectively). Similarly, there was no difference in the distribution of data among variables, between the young and elderly patients (Table 5). The rate of euthyroidism was higher among group 2 patients (≥65 years) as compared to group 1 (<65 years), 60% vs. 45% respectively. However, this difference was not statistically significant (Table 6).

**Figure 1:**
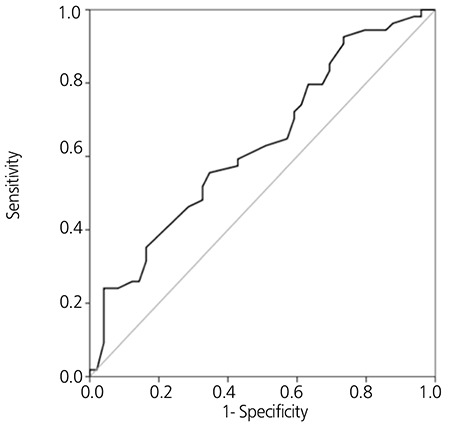
ROC curve of age. Area under the ROC curve of age was found as 0.632 (p=0.01), Cut off <=65, Sensitivity: 65.31%, Specificity: 55.56%

In the ROC curve analysis; the area under the curve was found as 0.632 (p=0.01) and the cut off was set as 65 years with 65, 31% sensitivity and 55% specificity (Figure 1); suggesting that the age effect on outcome was gaining significance after the age of 65.

## DISCUSSION

Ninety percent of patients were treated successfully with single dose RAI administration. At the end of the first year, the overall hypothyroidism rate was 30% and euthyroid state was achieved in 70% of patients. Age was the only variable that had a statistically significant effect on outcome among multiple variants. The mean age of euthyroid patients was found to be significantly higher than those who were not (p=0.01). In other words, a higher ratio of euthyroidism (60%) was achieved in the geriatric patient population in comparison to the younger group (45%). The difference between the rates of euthyroidism was not statistically significant (p=0.16), and we think this is related to the already high mean age of our patient population. We also determined that the effect of age on outcome was gaining significance after 65 years (Figure 1).

The rate of toxic nodular or multinodular goiter as etiologic factors increase with age. Ten to fifteen percent of hyperthyroid patients are over 60 years of age, and the incidence of nodular goiter is higher than its normal frequency in regions of low iodine intake such as our country. The incidence of nodular goiter increases up to 37.5% above 65 years of age in our country (2,3,4,5). This issue was evident in our patient population; the mean age of the patients in our study was 63±12.

The geriatric patient population is of particular importance because of its unique challenges. ATD medication has important side effects like rush, fever, arthralgia and dose limiting agranulocytosis, all of which are more frequent in the elderly (10,11). Surgical therapy has higher morbidity and mortality in the geriatric patient group. Due to these concerns, RAI therapy is the choice of treatment (2,3,11). The characteristic signs and symptoms of hyperthyroidism may be lacking, and some of these may be mistaken as an underlying malignancy or other chronic diseases. These challenges usually lead to a delay in diagnosis. Patients generally have cardiovascular co-morbidities at the time of diagnosis (3,10,11,12,13,14). For these reasons, hyperthyroidism in the elderly should be treated as soon as possible.

Dose protocols include the calculated dose, low empiric fixed dose, low and high fixed dose regimens (15). There is an ongoing argument on the choice of dose regimens in order to increase euthyroidism rates while reducing the total absorbed dose. However, the treatment success rates of toxic nodules with doses that are lower than 20 mCi were reported to be low (16). Additionally, many institutions advocate fixed dose regimens stating that it is not necessary to use dose calculation methods due to the additional time and cost requirement (17,18,19,20). RAI therapy in elderly patients should aim at rapid treatment of persistent hyperthyroidism; therefore, our department’s policy in such situations is administration of high fixed dose RAI therapy. Success rates up to 97% with 15 mCi fixed dose RAI therapy has been reported (21). Our study results are in accordance with these studies.

It was determined that hypothyroidism rates were relatively lower while euthyroidism rates did not seem to increase much in TNG patients treated with low fixed dose RAI therapy (5-10 mCi). Also an increase in persistent hyperthyroidism and the need of multiple RAI therapies is prominent (7,22,23). In our study, similar rates of hypothyroidism (30%) and treatment success (90%) were achieved with single dose administration.

An independent variable that effects treatment success and hypothyroidism rate was not identified in many studies (18,24,25,26,27). High rates of euthyrodism are expected after treatment of TNG with radiodine due to the relatively radioresistant nature of the nodules and the probable existence of different amounts of underlying suppressed thyroid tissue. Based on this fact, high fixed dose I-131 treatment regimen gained attraction. The experience of Freitas et al. (28) in 4at exclusively consisted of toxic autonomous functioning thyroid adenoma (TAFTA), and the possible higher amount of underlying suppressed thyroid tissue. In other words, 5 TNG patients who were treated with ≥10 mCi RAI therapy correlates with our single dose success rates (96% vs. 90%, respectively). However, their hypothyroidism rate was very low (8% vs. 30%). This may be explained with their patient population ththe rate of hypothyroidism after I-131 therapy is inversely related to the degree of RAI uptake by extra-nodular tissue (29).

Kang et al. (30) evaluated the outcome of TNG patients treated with surgery or high dose (mean 28 mCi) RAI therapy. The success rate of RAI therapy was stated as 98%. The resolution of hyperthyroidism was quicker in this study (mean 5.4 months) as compared to our study (mean 7 months). In addition, Kang et al. (30) reported a high rate of hypothyroidism (68%). These are expected results, since they administered a higher mean dose of RAI as compared to our study. Although rapid treatment of hypothyroidism is important, achieving a high rate of euthyroidism in our study is a significant gain.

Many patients with TNG are the elderly with concomitant illnesses; therefore, the goal of RAI therapy should be relieving hyperthyroidism with a single dose administration. We expect relatively high euthyroidism rates in geriatric TNG patients, due to the presence of underlying suppressed tissue and the decreased uptake by the aging thyroid. The higher ratio of euthyroidism in the geriatric patient group detected in our study supports this apparent event.

### Limitations

This retrospective study represents the routine treatment policy and results of our department. The I-131 uptake and thyroid gland measurements were not available for analysis. Nevertheless, long-term follow-up results of a homogenous subgroup of patients undergoing RAI treatment reflects ten years of experience of a university hospital nuclear medicine department.

## CONCLUSION

High fixed dose I-131 treatment should be preferred in geriatric TNG patients in order to treat persistent hyperthyroidism rapidly due to systemic co-morbidities. The results of this study suggest that high fixed dose RAI therapy is a successful modality in treating TNG and higher euthyroidism rates can be achieved in geriatric patients.

## Figures and Tables

**Table 1:**
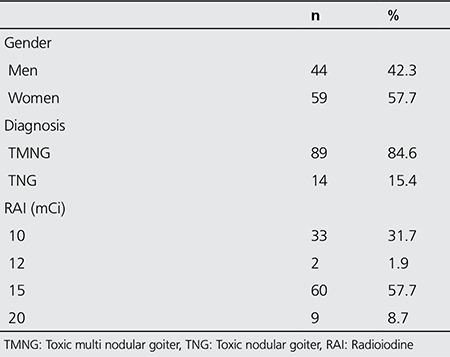
Patient population

**Table 2:**
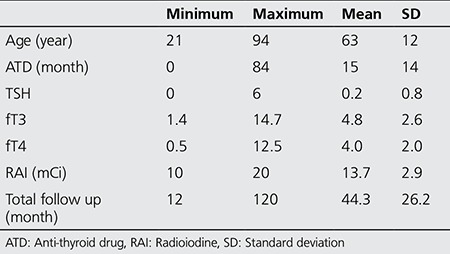
Patient characteristics

**Table 3:**
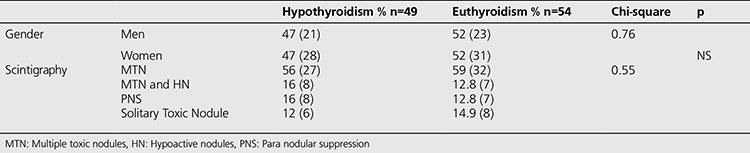
Effect of gender and scintigraphic pattern on outcome

**Table 4:**
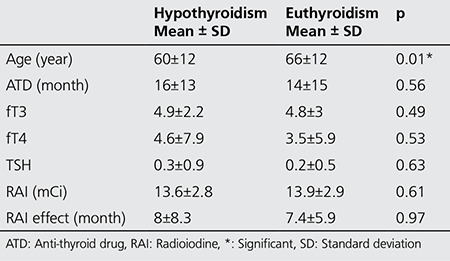
Effect of different factors on outcome

**Table 5:**
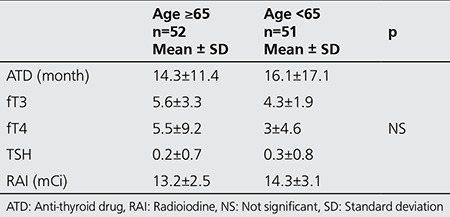
Characteristics of the younger and geriatric patients

**Table 6:**
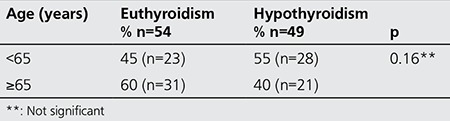
Effect of age on outcome

## References

[1] Thomas CG, Croom RD (1987). Current management of the patient with autonomously functioning nodular goiter. Surg Clin North Am.

[2] Kennedy JW, Caro JF (1996). The ABCs of managing hyperthyroidism in the older patient. geriatrics.

[3] Diez JJ (2003). Hyperthyroidism in patients older than 55 years: An analysis of the etiology and management. Gerontology.

[4] Erdoğan G, Erdogan MF, Emral R, Baştemir M, Sav H, Haznedaroğlu D, Ustündağ M, Köse R, Kamel N, Genç Y (2002). Iodine status and goiter prevalence in Turkey before mandatory iodization. J Endocrinol Invest.

[5] Erdogan MF, Deni O, Gursoy A (2012). Tiroid nodüllerine yaklasım. İçinde: A dan Z ye klinik tiroidoloji. İstanbul, Omur Matbaacılık.

[6] Kalinyak JE, McDouggall IR (2003). How should the dose of iodine-131 be determined in the treatment of Graves’ hyperthyroidism?. J Clin Endocrinol Metab.

[7] Allahabadia A, Daykin J, Sheppard MC, Gough SC, Franklyn JA (2001). Radioiodine treatment of hyperthyroidism- prognostic factors for outcome. J Clin Endocrinol Metab.

[8] Helol KD, Harbert JC (1987). Radiobiology: biologic effects of ionizing radiations. Nuclear medicine therapy.

[9] Lombardi MH, Lombardi MH (1999). Unites of radiation exposure and dose. Radiation safety in nuclear medicine.

[10] Martin FI, Dream DR (1996). Hyperthyroidism in elderly hospitalised patients. Clinical features and treatment outcomes. Med J Aust.

[11] Tajiri J, Noguchi S (2004). Antithyroid drug-induced agronulocytosis: special reference to normal white blood cell count agranulocytosis. Throid.

[12] Solomon B, Glinoer D, Lagasse R, Wartofsky L (1990). Current trends in the management of Graves disease. J Clin Endocrinol Metab.

[13] Stefano M, Cladio F, Andrea C, Aldo P (1995). The Aging Thyroid. Endocrine Reviews.

[14] Aronow WS (1995). The heart and thyroid disease. Clin Geriatr Med.

[15] (14.02.2002). EANM Procedure Guideline for therapy with I-131, revised. growth.

[16] Greenspan FS, In: Basic&clinical endocrinology (1997). The thyroid gland. Appleton and Lange.

[17] Kinser JA, Roesler H, Furrer T, Grutter D, Zimmerman H (1989). Nonimmunogenic hyperthyroidism: cumulative hypothyroidism incidence after radioiodine and surgical treatment. J Nucl Med.

[18] Jarlov AE, Hegedüs L, Kristensen LO, Nygaard B, Hansen B (1995). Is calculation of the dose in radioiodine therapy of hyperthyroidism worth wile?. Clin Endocrinol Metab.

[19] Nordyke RA, Gilbert FI (1991). Optimal iodine-131 dose for eliminating hyperthyroidism in Graves’ disease. J Nucl Med.

[20] Gittoes NJ, Franklyn JA (1998). Hyperthyroidism. Current treatment guidelines: Drugs.

[21] Tang Fui SC, Maisey MN (1979). Standard dose-131l therapy for hyperthyroidism caused by autonomously functioning thyroid nodules. Clin Endocrinol (Oxf).

[22] Metso S, Jaatinen P, Huhtala H, Luukkaala T, Oksala H, Salmi J (2004). Long-term follow-up of radioiodine treatment of hyperthyroidism. Clin Endocrinol (Oxf).

[23] Tarantini B, Ciuoli C, Cairano G, Guarino E, Mazzucato P, Montanaro A, Burroni L, Vattimo AG, Pacini F (2006). Effectiveness of radioiodine (131-I) as definitive therapy in patients with autoimmune and non-autoimmune hyperthyroidism. J Endocrinol Invest.

[24] Vijayakumar V, Ali S, Nishino T, Nusynowitz M (2006). What influences early hypothyroidism after radioiodine treatment for Graves’ hyperthyroidism?. Clin Nucl Med.

[25] Turner J, Sadler W, Brownlie B, Rogers T (1985). Radioiodine therapy for Graves’ disease; multivariate analysis of pretreatment parameters and early outcome. Eur J Nucl Med.

[26] Catargi B, Leprat F, Guyot M, Valli N, Ducassou D, Tabarin A (1999). Optimized radioiodine therapy of Graves’ disease: analysis of the delivered dose of other possible factors affecting outcome. Eur J Endocrinol.

[27] Leslie WD, Ward L, Salamon EA, Ludwig S, Rowe RC, Cowden EA (2003). A randomized comparison of radioiodine doses in Graves’ hyperthyroidism. J Clin Endocrinol Metab.

[28] Freitas JE (2000). Therapeutic Options in the management of toxic and nontoxic nodular goiter. Semin Nucl Med.

[29] Mariotti S, Martino E, Francesconi M, Ceccarelli C, Grasso L, Lippi F, Baschieri L, Pinchera A (1986). Serum thyroid autoantibodies as a risk factor for development of hypothyroidism after radioactive iodine therapy for single thyroid ‘hot’ nodule. Acta Endocrinol (Copenh).

[30] Kang AS, Grant CS, Thompson GB, Heerden JA (2002). Current treatment of nodular goiter with hyperthyroidism (Plummer’s disease): surgery versus radioiodine. Surgery.

